# Aerosolized Zika virus infection in Guinea pigs

**DOI:** 10.1080/22221751.2022.2122577

**Published:** 2022-09-29

**Authors:** Hong-Ying Qiu, Na-Na Zhang, Qing-Qing Ma, Rui-Ting Li, Meng-Yue Guan, Li-Li Zhang, Jia Zhou, Rong-Rong Zhang, Xing-Yao Huang, Wen-Hui Yang, Yong-Qiang Deng, Cheng-Feng Qin, Dong-Sheng Zhou

**Affiliations:** a State Key Laboratory of Pathogen and Biosecurity, Beijing Institute of Microbiology and Epidemiology, Beijing, People’s Republic of China; b School of Medicine, Tsinghua University, Beijing, People’s Republic of China; c Beijing Traditional Chinese Medicine Hospital, Capital Medical University, Beijing, People’s Republic of China

**Keywords:** Zika virus, aerosol, transmission, Guinea pig, animal model

## Abstract

Zika virus (ZIKV) is primarily transmitted through mosquito bites and sexual contact, and vertical transmission of ZIKV has also been observed in humans. In addition, ZIKV infection via unknown transmission routes has been frequently reported in clinical settings. However, whether ZIKV can be transmitted via aerosol routes remains unknown. In this study, we demonstrated that aerosolized ZIKV is fully infectious in vitro and in vivo. Remarkably, intratracheal (i.t.) inoculation with aerosolized ZIKV led to rapid viremia and viral secretion in saliva, as well as robust humoral and innate immune responses in guinea pigs. Transcriptome analysis further revealed that the expression of genes related to viral processes, biological regulation and the immune response was significantly changed. Together, our results confirm that aerosolized ZIKV can result in systemic infection and induce both innate and adaptive immune responses in guinea pigs, highlighting the possibility of ZIKV transmission via aerosols.

## Introduction

Zika virus (ZIKV) is a mosquito-borne *flavivirus* in the *Flaviviridae* family, which includes a large number of pathogens, such as dengue virus (DENV), West Nile virus (WNV), Japanese encephalitis virus (JEV), yellow fever virus (YFV), and tick-borne encephalitis virus (TBEV) [1]. Flaviviruses have single positive-stranded 11-kb RNA genomes in which the 5’ and 3’ untranslated regions flank a polyprotein coding region encoding three structural proteins [capsid, pre/membrane (prM), and envelope (E)] and seven nonstructural proteins (NS1, NS2A, NS2B, NS3, NS4A, NS4B, and NS5). Although most Zika infections are asymptomatic or present with benign, self-limited symptoms, a small percentage of patients have complications, such as congenital anomalies in the developing foetus of pregnant women infected with the virus and neurological complications (Guillain‒Barré syndrome). To date, there is no vaccine, antiviral drug, or other modality available to prevent or treat Zika virus infection [2].

Similar to other mosquito-borne flaviviruses, ZIKV is primarily transmitted to humans through bites by mosquito vectors. However, human-to-human transmission has also been reported for ZIKV via blood transfusion, sexual contact, and transplacental transmission [3–6]. Moreover, infectious ZIKV can be present in saliva, urine, semen, and breast milk [7–10], and transmission via the skin or oronasal mucosa has been observed in humans and experimental animals [1,11,12]. Interestingly, a previous study suggested that DENV could be isolated from the upper respiratory tract of patients with dengue fever[13]. Another study also indicated that Tembusu virus (TMUV), a *flavivirus* related to ZIKV, could be transmitted efficiently among ducks by aerosol transmission [14]. Furthermore, JEV was isolated from the nasal secretions of pigs [15], and transmission has been demonstrated to occur between animals via aerosols [16]. However, whether ZIKV can infect and be transmitted via aerosols remains unknown.

Guinea pigs have been widely used as a well-established animal model to evaluate ZIKV infection and pathogenesis [1,17–19]. Our previous study indicated that guinea pigs were susceptible to ZIKV infection via subcutaneous or intranasal inoculation routes[1]. Herein, we further applied aerosolized ZIKV directly via intratracheal (i.t.) inoculation to the lungs of guinea pigs to assess the risk of aerosol-mediated ZIKV infection. The results indicated that aerosolized ZIKV can cause systemic infection and induce both innate and adaptive immune responses in guinea pigs.

## Material and methods

### Ethics statement

All animal experiments were performed according to the guidelines of the Chinese Regulations of Laboratory Animals (Ministry of Science and Technology of People’s Republic of China). All animal procedures were approved by the Animal Experiment Committee of the Laboratory Animal Center, AMMS, China (approval number: IACUC-IME-2021-010).

### Cells and viruses

BHK-21 cells (#CCL-10) were purchased from ATCC and cultured in Dulbecco's modified Eagle medium (DMEM) containing 10% foetal bovine serum (FBS), 100 U/mL penicillin and 100 g/ml streptomycin. C6/36 cells (ATCC# CRL-1660) were cultured in RPMI-1640 containing 10% FBS, 100 U/mL penicillin and 100 g/mL streptomycin. Guinea pigs in this study were inoculated with the ZIKV GZ01 strain (GenBank accession no: KU820898), which was originally isolated from a Chinese patient who returned from Venezuela in 2016 [7]. The viral stocks used in this study were prepared on a confluent monolayer of mosquito C6/36 cells, titrated in BHK-21 cells for the plaque-forming assay, and stored at −80 °C.

### Aerosolization of ZIKV solution

Aerosolization of ZIKV solution was performed using a hand-held liquid aerosol pulmonary delivery device (HLAPDD) (Huironghe Company, Beijing, China) suitable for guinea pigs. The device is an aerosol generator that ejects aerosol solutions directly into the lungs and achieves precise quantification. The tip of the device was inserted into a 1.5-ml EP tube with a hole, and 50 μl of ZIKV aerosol was generated. The volume was measured with a pipette after centrifugation. The particle size of individual aerosols was measured using an aerodynamic particle sizer (APS 3321, TSI, USA) with a sampling flow rate of 5 L/min and time of 15 s. The mean mass aerodynamic diameter (MMAD) of the aerosol particles was determined by Aerosol Instrument Manager Software.

### In vitro phenotypes of aerosolized ZIKV

Briefly, ZIKV aerosols were collected using a 1.5-ml EP tube and then centrifuged for 1 min at 8000 r/min for subsequent RT‒qPCR, immunofluorescence staining and plaque assays, and the replication curve, virus protein expression and virus titre were compared with those of the original solution.

For the replication curve, BHK-21 cells were transferred to 24-well plates and cultured at 37 °C with 5% CO2. Then, the liquid and aerosolized Zika virus were added to BHK-21 cells and incubated at 37 °C for 1 h. Infected cells were cultured at 37 °C with 5% CO2, and the cell supernatant was collected at 24, 48, and 72 h post-infection (hpi). The viral RNA load in the cell supernatant was determined by RT‒qPCR, and the replication curve of the liquid and aerosolized Zika virus on BHK-21 cells was plotted by GraphPad Prism 8 software.

For viral protein expression, the infected cells were fixed with methanol/acetone (v/v: 7/3) at 48 h post-infection. The cells were then treated with the anti-Zika ENV mAb (Cat No. BF-1176-56, 1:1000 diluted, BioFront Technologies) and incubated at 37 °C for 1 h. Goat anti-mouse IgG-Alexa Fluor 488 (1:200 diluted, Gene-Protein Link) was added, and the plates were incubated at 37 °C for 1 h. For cell nucleus staining, 4,6-diamidino-2-phenylindole (DAPI, 0.5 ng/μL) was added to the wells, and the plates were incubated for 5 min. An Olympus IX73 microscope was used for image acquisition.

For determination of the virus titre, BHK-21 cells were cultured in a 12-well plate for 24 h and then infected with 500 µL of 10-fold viral dilutions for 2 h at 37 °C. Viral supernatants were replaced with DMEM containing 1% low melting point agarose (Promega) and 2% FBS. At 4 days after infection, the cells were fixed with 4% formaldehyde, followed by staining with 1% crystal violet solution. Finally, all visible plaques were counted, and the final titres were calculated as plaque-forming units/mL (PFU/mL).

### Aerosol distribution

The guinea pigs were sacrificed by exposure to CO2. Aniline blue solution was injected directly into the lung, and then the lung was dissected to verify the distribution of aerosols and photographed. To further verify the homogeneity and specificity of aerosol distribution in the lung, Near-infrared fluorescent Degradex® poly microspheres (Hopkinton, MA, USA) were sprayed into the lung via i.t. inoculation using a HLAPDD. Briefly, guinea pigs were anaesthetized with pentobarbital sodium by intraperitoneal injection. A special support equipped with a nylon wire was used to suspend the guinea pigs by their upper incisors at 60°. Optimal illumination of the trachea was achieved by a laryngoscope (Huironghe Company, Beijing, China). After a clear view of the trachea was obtained, the tip of the device was inserted, and 50 µl of aerosol was sprayed into the lung by depressing the syringe piston with a constant force [20]. The guinea pig was sacrificed 24 h after administration, and the organs were imaged using an IVIS Spectrum small-animal imaging system (λex: 745 nm; λem: 820 nm; exposure time: 10 s; binning factor, f-stop 2; field of view: D).

### Animal experiments

Male guinea pigs (Hartley strain) weighing 280–300 g were purchased from Charles River Laboratories and inoculated with 10^5^ PFU of ZIKV by the i.t. route using a HLAPDD. Serum and saliva samples were collected and processed at 1, 2, 3, 5 and 7 days post-infection (dpi) as described previously [1]. The viral loads of the samples were analysed by quantitative reverse transcription PCR (RT‒qPCR) and are expressed as RNA copies per millilitre. Guinea pigs were euthanized by exposure to CO2 at 1, 2, 3 and 7 dpi to isolate tissues. At 2 or 7 dpi, the viral loads of various tissues were detected, and lung tissues were collected for subsequent histopathology assays, immunofluorescence staining and RNA ISH assays as described below. Transcriptome analysis of the lungs was conducted at 1, 3 and 7 dpi. Cellular immune responses in serum were assessed at 1, 3, 5 and 7 dpi. Serum was collected at 7, 14, 21 and 28 dpi for the detection of ZIKV-specific IgG as described below.

### Viral load assay

The viral RNA was extracted from 0.2 ml of saliva, the supernatants of tissues, the BioSampler collection medium and 0.1 ml of serum using a PureLink® RNA Mini Kit (Life Technology, USA) according to the manufacturer’s instructions and eluted in 60 μl of RNase-free water. RT‒qPCR was performed using the One Step PrimeScript RT‒PCR Kit (Takara, Japan) with the primers and probe as described previously [1]. RNA copies per ml or RNA copies per gram were calculated from quantitative PCR Ct values with a published method [21].

### Histopathology assay

Following experimental ZIKV aerosol infection of guinea pigs, euthanasia was conducted at the indicated times. Lung tissues were fixed in 4% neutral buffered formalin and embedded in paraffin. The samples were cut into 5 μm thick sections, and after rehydration with a gradient, they were stained with haematoxylin and eosin. Images were captured using an Olympus BX51 microscope equipped with a DP72 camera. The original magnification was 20×.

### Immunofluorescence staining

For immunostaining, lung tissues were fixed with 4% paraformaldehyde for 24 h at 4 °C and then dehydrated in 30% sucrose for 24 h. The slices were blocked at room temperature (RT) for 1 h in 3% bovine serum albumin, 10% FBS, and 0.2% Triton X-100 in PBS, incubated with mouse anti-ZIKV E protein antibody (BF-1176-56, BioFront, China) overnight at 4 °C, washed with 0.2% Triton X-100 in PBS (3×10 min), incubated in fluorescein isothiocyanate-conjugated goat anti-mouse IgG (ZSGB-Bio, China) at RT for 1 h, and then washed three times. The nuclei were stained with 4’,6-diamidino-2-phenylindole (DAPI, Invitrogen).

### RNA ISH assay

The ZIKV genomic RNA ISH assay was performed with an RNAscope kit (Advanced Cell Diagnostics, USA) according to the manufacturer’s instructions. Briefly, formalin-fixed paraffin-embedded (FFPE) tissue samples were deparaffinized with xylene and incubated with hydrogen peroxide for 10 min at room temperature to neutralize endogenous peroxidases. The samples were boiled in RNAscope Target Retrieval Reagent for 15 min, followed by incubation in RNAscope Protease Plus for 30 min and probe hybridization for 2 h. The signal was amplified and visualized by using the Brown (DAB) Detection Kit. Tissues were counterstained with 50% Gill’s haematoxylin and visualized with standard bright-field microscopy. The original magnification was 20×.

### ZIKV-specific antibody detection

Serum IgG antibodies against ZIKV were detected by ELISA. Briefly, polysorb enzyme-linked immunosorbent assay plates (Nunc, USA) were coated at 50 ng per well with ZIKV prME protein (Sino Biological, China) diluted in phosphate buffered saline (PBS) overnight at 4 °C. Plates were blocked in 5% (vol/vol) bovine serum albumin in PBS for 1 h at 37 °C. Serum samples were serially diluted in PBS and added to the wells, and the plates were incubated for 1 h at 37 °C. The plates were then washed with PBST five times to remove unbound antibody. After washing, a suitable concentration of guinea pig IgG antibody (Abcam, UK) was added to the well for 1 h at 37 °C, TMB substrate (Solarbio, China) was added to the wells, and the plate was incubated for 20 min at room temperature in darkness. Finally, 2.0 M H_2_SO_4_ was added to stop the reaction, and the absorbance was measured at 450 nm using a microplate reader (Beckman, USA). The ZIKV E-specific antibody titre was calculated based on the highest dilution that resulted in a value twofold greater than the absorption of the control serum, with a cut-off value of 0.05.

### Measurement of cytokines and chemokines

The concentrations of cytokines and chemokines were measured in the serum collected from guinea pigs by a commercial Luminex immunoassay kit (Cytokine & Chemokine 22-Plex Rat ProcartaPlex™ Panel, Thermo Fisher Scientific, Germany) according to the manufacturer’s instructions.

### RNA library construction and sequencing

Guinea pigs before or after infection (1, 3 or 7 dpi) as previously described were used for RNA-seq. Total RNA from lung tissues was extracted using TRIzol (Invitrogen, USA) and DNase I (NEB, USA), respectively. Sequencing libraries were generated using the NEBNext^®^ Ultra^TM^ RNA Library Prep Kit for Illumina^®^ (#E7530L, NEB, USA) following the manufacturer’s recommendations, and index codes were added to attribute sequences to each sample. Clustering of the index-coded samples was performed on a cBot cluster generation system using a TruSeq PE Cluster Kit v3-cBot-HS (Illumina, San Diego, California, USA) according to the manufacturer’s instructions. After cluster generation, the libraries were sequenced on the Illumina NovaSeq 6000 platform, and 150-bp paired-end reads were generated. After sequencing, a Perl script was used to filter the original data (raw data) to clean reads by removing contaminating reads with adapter sequences and low-quality reads. Clean reads were aligned to the *Cavia porcellus* genome (Cavpor3.0). The number of reads mapped to each gene in each sample was counted by featureCounts v1.5.0-p3, and the FPKM (transcripts per kilobase of exon model per million mapped reads) was then calculated to estimate the expression levels of genes in each sample.

### Bioinformatic analyses

The DESeq2 R package was used for differential gene expression analysis. Genes with Padj ≤ 0.05 and |Log2FC | > 1 were identified as differentially expressed genes (DEGs). The DEGs identified were used as queries to search for enriched biological processes (Gene Ontology BP) and KEGG pathway enrichment using Metascape. Heatmaps of gene expression levels were constructed using the pheatmap package in R (https://cran.rstudio.com/web/packages/pheatmap/index.html).

### Quantification and statistical analysis

All data were analysed with GraphPad Prism 8.0 software. Unless specified, the data are presented as the mean ± SEM in all experiments. Analysis of variance (ANOVA) or a t test was used to determine statistical significance among different groups (*P< 0.05; **P< 0.01; ***P< 0.001; ****P< 0.0001; n.s., not significant).

## Figures and Tables

**Figure 1.**
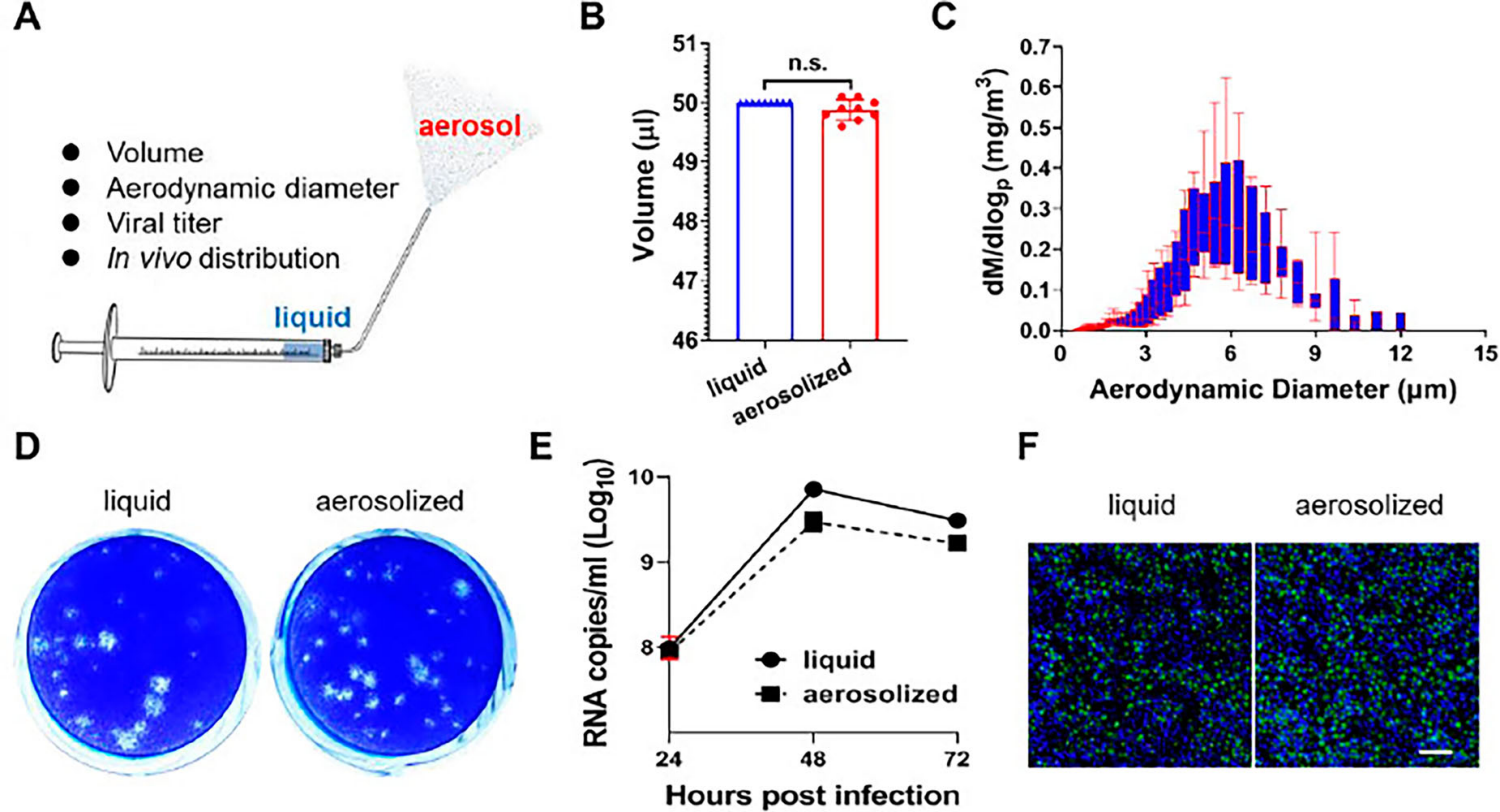
Biological characteristics of Zika virus aerosolization. (A) Schematic diagram of the hand-held liquid aerosol pulmonary delivery device (HLAPDD). (B) Volume comparison between pre- and postaerosolization ZIKV. (C) Representative aerodynamic median mass diameter (MMAD) graph of Zika virus aerosolization by an aerodynamic particle size spectrometer. (D) The viral titres of pre- and postaerosolization ZIKV. Briefly, an HLAPDD was used to aerosolize ZIKV. The tip of the device was inserted into a 1.5-ml EP tube with a hole, and 200 μl of ZIKV aerosol was generated. The EP tube was left for 30 min and centrifuged for 30 s at 8000×g to collect the sample for the plaque assay. (E) Replication curve of pre- and postaerosolization ZIKV in BHK-21 cells. Briefly, BHK-21 cells were infected with liquid and aerosolized ZIKV, and cell supernatants were collected at 24, 48, and 72 hpi to detect the viral RNA load by the RT–qPCR assay. (F) Viral protein expression of pre- and postaerosolization ZIKV in BHK-21 cells. Briefly, BHK-21 cells were infected with liquid and aerosolized ZIKV, and then the cells at 48 hpi were fixed for immunofluorescence staining. The E protein was stained green, and DAPI (blue) staining indicated the nucleus. Scale bar: 100 μM.

**Figure 2.**
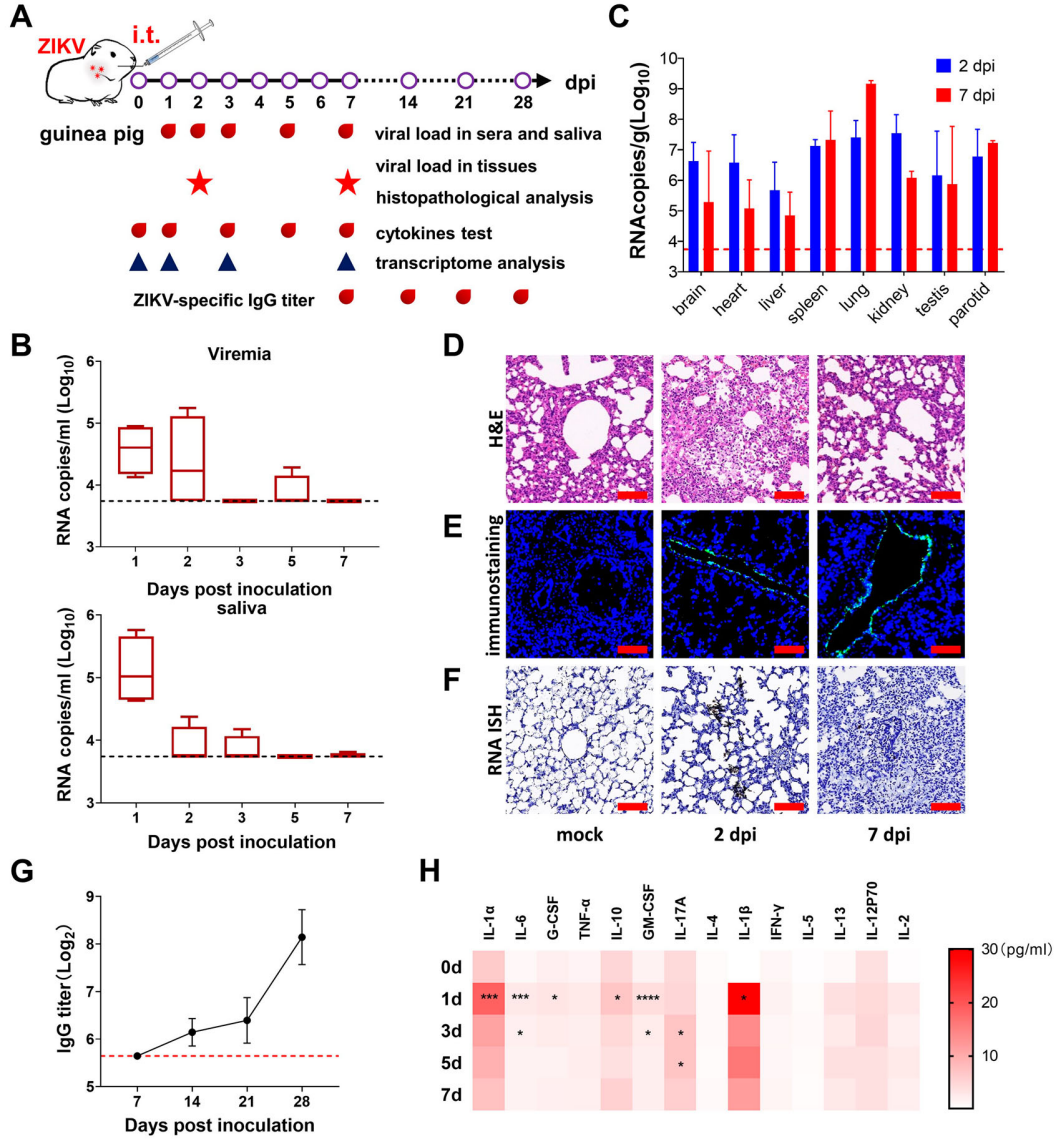
Guinea pigs were susceptible to ZIKV aerosol infection and triggered ZIKV-specific antibody and inflammatory responses. (A) Schematic diagram of the experimental design. Briefly, groups of guinea pigs were infected with 10^5^ PFU of ZIKV via i.t. inoculation, and the virological, pathological, immunological response and transcriptome analysis were characterized at the indicated times. (B) Guinea pigs were inoculated ZIKV aerosols via i.t. inoculation, and viral RNA loads in the serum and saliva were determined by RT–qPCR at the indicated times. The viral load is expressed as RNA copies per millilitre. Dotted lines indicate the limit of detection. Whiskers: 5–95 percentile. (C) Tissue distribution of ZIKV RNAs in the i.t. inoculated guinea pigs at 2 and 7 dpi. The data are shown as the mean±SD. (D) Histopathological changes in the lungs. All tissue samples were collected from ZIKV-infected guinea pigs at 2 and 7 dpi. Scale bar: 100 μm (E) Immunofluorescence staining of lung tissues was performed with a primary antibody against ZIKV E protein at 2 and 7 dpi. Nuclei were stained with DAPI. Scale bar: 100 μm. (F) ISH assay of ZIKV RNA at 2 and 7 dpi. Positive signals are shown in brown. Scale bar: 100 µm. (G) Specific IgG ZIKV E protein antibody titres in guinea pigs (n = 4) were determined by ELISA. IgG antibody titres were calculated according to the highest dilution that resulted in a value twofold greater than the absorption of the control serum. The data are shown as the mean ± SD. (H) Cytokine levels in ZIKV-infected guinea pigs in serum: Serum was collected at the indicated times. Data are shown as the mean ± SEM (n = 4). Significance was calculated using one-way ANOVA (n.s., not significant; *, *P* < 0.05; ***, *P* < 0.001; ****, *P*<0.0001).

**Figure 3.**
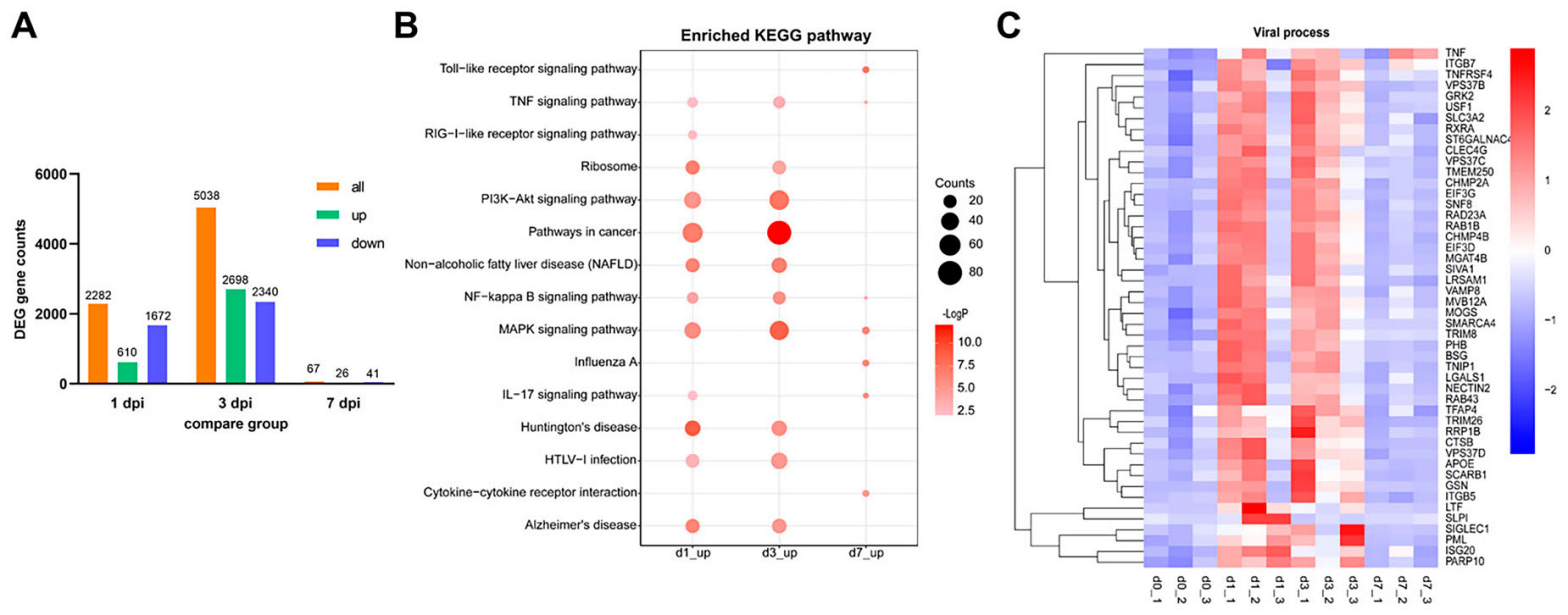
Differentially expressed genes (DEGs) in a guinea pig model of aerosolized ZIKV infection derived by RNA-seq. (A) The number of DEGs at different times after aerosol infection. (B) Visualization of enriched KEGG pathways of upregulated genes at 1, 3 and 7 dpi in the lungs. Gene enrichment analyses were performed using Metascape against the KEGG dataset. The heatmap cells are coloured according to their *p* values. (C) Heatmap indicating the expression patterns of 48 genes related to “viral process” that were significantly upregulated at 1 or 3 dpi. The coloured bar represents the Z score of the FPKM.

## Data Availability

All sequence data generated by RNA-seq have been deposited in the GEO database (accession no. GSE210370). All relevant data from this study are available from the authors upon request.

## Results

### Aerosolized ZIKV remains infectious in vitro

The biological activities of ZIKV aerosolized using a hand-held liquid aerosol pulmonary delivery device (HLAPDD) [22] were determined (Figure 1A). First, volumes pre- and postaerosolization of ZIKV were measured and showed no statistically significant differences (Figure 1B). The particle sizes of aerosolized ZIKV were calculated by using the time-of-flight principle, and the mean mass aerodynamic diameter (MMAD) was 5.34±0.05 μm (Figure 1C), which was approximately consistent with the distribution range in the lung (1-5 μm). More importantly, we compared the in vitro phenotypes of the liquid and aerosolized ZIKV in BHK-21 cells by using RT‒qPCR, immunofluorescence and plaque assays. The results of the plaque assays indicated that aerosolized ZIKV infection produced a homogeneous small-plaque phenotype in BHK-21 cells similar to that of liquid ZIKV infection (Figure 1D). The RT‒qPCR results further showed that the liquid and aerosolized ZIKV infections exhibited consistent kinetics of replication in BHK-21 cells without significant differences, and the peak titre reached ∼10^9^ RNA copies/mL at 48 hpi (Figure 1E). Finally, IFA results also showed that E protein-specific proteins were detected in both the liquid and aerosolized ZIKV-infected BHK-21 cells at 48 hpi (Figure 1F). These results suggested that aerosolized ZIKV remains infectious in vitro.

To evaluate the in vivo aerosolization effect of HLAPDD, 6∼8-week-old guinea pigs were i.t. inoculated with aniline blue solution and near-infrared fluorescent microspheres by HLAPDD, and the distribution in lung and other organs, respectively, was observed. The results clearly indicated that the aniline blue solution was distributed uniformly in the lungs at 2 h after the i.t. inoculation (**Figure S1**). As expected, the results from the IVIS Spectrum imaging system also indicated that the fluorescence was distributed evenly over the lung lobes at 24 h after i.t. inoculation, and the other organs had no fluorescent deposition (**Figure S2**).

### Aerosolized ZIKV via intratracheal inoculation leads to systemic infection in Guinea pigs

To further assess the infectivity of aerosolized ZIKV in guinea pigs, groups of 3∼4-week-old male guinea pigs were subjected to i.t. inoculation with 10^5^ PFU of ZIKV, and the virological, pathological, immunological responses and transcriptome were characterized in detail (Figure 2A). The results indicated that sustained viremia and robust viral RNA shedding in saliva were detected in all animals from 1 to 5 days post-infection, with a peak titre of 5.8 log RNA copies/ml in saliva at 1 dpi as well as peak titres of 5.2 log RNA copies/ml in viremia at 2 dpi (Figure 2B). These results are similar in magnitude and pattern compared with those for ZIKV infection in a guinea pig model via the s.c. route or i.n. route [1].

Then, necropsy was performed at 2 and 7 dpi, and high levels of ZIKV RNAs were detected in multiple organs, including the lung, brain, spleen, kidney and parotid gland, at 2 dpi. Notably, the viral load in the lung increased significantly, with a peak titre of 9.2 log RNA copies/g at 7 dpi (Figure 2C). Specifically, histopathology suggested that ZIKV infection resulted in substantial pathological changes in the lung at 2 and 7 dpi, including alveolar structural damage accompanied by slight inflammatory cell infiltration and haemorrhage (Figure 2D). Immunostaining further indicated that robust viral E protein was detected along the airway of the lungs at 2 and 7 dpi (Figure 2E). The ISH assay by RNAscope also showed that ZIKV-specific RNA was detected in the lungs (Figure 2F). Specifically, ZIKV-specific IgG antibodies were detected at 14 dpi, with levels reaching 1/300 at 28 dpi (Figure 2G). Additionally, the levels of multiple cytokines, including IL-1α, IL-6, GM-CSF, IL-1β, IL-10 and G-CSF, increased significantly at 1 and 3 dpi. The level of IL-17A increased significantly at 3 and 5 dpi (Figure 2H). These results demonstrated that aerosolized ZIKV can cause systematic infection and induce both innate and adaptive immune responses in guinea pigs.

### Aerosolized ZIKV infection triggers innate immune responses in the lungs

To further explore the molecular responses to aerosolized ZIKV infection, we examined the transcription level in lungs at 1, 3 and 7 days after infection. The results showed changes in the expression of numerous genes and multiple antiviral processes and activated immune pathways compared with the uninfected animals. Specifically, the number of genes with upregulated or downregulated expression increased at 1 dpi, and peak DEG expression was detected at 3 dpi, followed by a rapid decrease at 7 dpi. There were 2698 upregulated and 2340 downregulated DEGs detected at 3 dpi. A total of 26 genes were upregulated and 41 genes were downregulated at 7 dpi (Figure 3A). This result was consistent with the trend for the viral load in serum and saliva. A Venn diagram was plotted based on all of the DEGs at 1, 3 and 7 dpi. There were 1913 shared DEGs between 1 and 3 dpi, whereas there were only 17 shared DEGs among all three time points, as shown **in Figure S2**. The number of specific DEGs was greater at 3 dpi than at the other time points. Moreover, comparison of KEGG pathways showed that ZIKV infection was associated with multiple pathways, including NF-kappa B signalling, the MAPK signalling pathway, PI3K-Akt signalling, Th-17-cell differentiation signalling, the Toll-like receptor signalling pathway, and other immune pathways (Figure 3B). Furthermore, Gene Ontology analysis at the biological process level revealed that genes related to viral processes were significantly enriched at 1 and 3 dpi. Of all significantly upregulated genes, 48 were involved in viral processes, including regulation of viral assembly and budding, as well as defence responses to viruses (Figure 3C).

## Discussion

Here, we established an aerosolized ZIKV-infected guinea pig model via the i.t. route. Our results showed that aerosolized ZIKV can cause viremia and viral shedding in saliva at 1–5 dpi, with peak titres of 5.2 log RNA copies/ml in serum at 2 dpi. However, previous results indicated that ZIKV infection is related to virus strains, guinea pig strains, inoculation levels and infection routes [1,17–19]. For instance, after subcutaneous inoculation with the Zika virus PRVABC59 strain, the viral load in serum peaked at 2 dpi, decreased at 3 dpi, and was undetectable at 5 dpi [17]. Similarly, guinea pigs developed persistent viremia on days 2–5 after subcutaneous infection with the Zika virus GZ01 strain, with a peak (5.4–6.9 log RNA copies/ml) observed at 3 dpi. Simultaneously, typical viral shedding kinetics in serum and saliva were observed in animals with intranasal infection [1]. In addition, the viral load in vaginal lavage of guinea pigs peaked at 3 dpi (425 PFU) after vaginal inoculation with the MR766 strain of Zika virus, and low levels of viral RNA were detected in serum and were sustained for up to 37 dpi [18]. In contrast, strain 13 guinea pigs inoculated intraperitoneally with ZIKV strain ArD 41525 or strain CPC-0740 did not have detectable viremia, and pathological changes were not observed in multitissue organs [19].

Meanwhile, our results also indicated that viral RNA was detected in multiple tissues at 2 and 7 dpi, and the viral load in the spleen and parotid gland increased at 7 dpi. A previous study showed that viral RNA was detected in the multitissue organs of rhesus macaques at 5 dpi with Zika virus infection and with a higher viral load in the parotid glands and spleen [21]. Another study showed that viral RNA was detected in the spleens of vaginally inoculated infected guinea pigs, and a higher copy number of ZIKV was detected from 3 dpi to 37 dpi [18]. Specifically, our results indicated that lung tissue had the highest viral load of all tissues at 7 dpi, which may be closely related to the route of infection that we used.

Subsequently, our results also indicated that the expression of multiple inflammatory factors was upregulated after i.t. infection. Specifically, IL-1β and IL-6 expression was significantly increased at 1 dpi, and IL-6 sustained the high expression level up to 3 dpi, which suggests that IL-1β plays a crucial role in the response to infection by Zika virus [23] as well as other flaviviruses, WNV [24] and JEV [25]. Recent studies reported that the inflammatory mediator IL-1β was the top cerebrospinal fluid marker in both microcephalic and nonmicrocephalic cases exposed to ZIKV [23], and increased levels of IL-1β and IL-6 chemokines were detected in ZIKV-infected human neural progenitor cells. Similarly, another result indicated that IL-6 expression was upregulated at 1 dpi and significantly elevated at 2 and 3 dpi, which was correlated with cell death [26]. Importantly, ZIKV-specific IgG antibodies were detected at 14 dpi, and the level reached 1/300 at 28 dpi, which is consistent with our previous result after s.c. or i.n. infection (Figure 2G). These combined findings clearly indicate that ZIKV aerosols could effectively replicate in the lungs, resulting in lung damage and systematic infection.

Finally, we investigated the transcriptome profiles of the lungs of guinea pigs in response to ZIKV aerosol infection at 1, 3, and 7 dpi using transcriptome sequencing. Fortunately, the results of KEGG pathway enrichment revealed that multiple signalling pathways were enriched in the lungs, including the MAPK signalling pathway, PI3K/Akt signalling pathway and NF-kappa B signalling pathway. These results were consistent with those for several ZIKV-infected nervous system cells [27–31]. For example, the MAPK signalling pathway has been shown to be dysregulated in ZIKV-infected neural progenitor cells and Sertoli cells and may be needed for ZIKV replication [27,32–34]. Another study also showed that ZIKV infection led to increased transcript levels of proinflammatory cytokines such as IL-1β and IL-6 by activating NF-kappa B signalling [35]. In our study, the levels of the cytokines IL-1β and IL-6 were significantly increased at 1 dpi, and the high expression level of the cytokine IL-6 was sustained at 3 dpi (Figure 2H). Meanwhile, the PI3K/Akt signalling pathway is enriched after ZIKV infection, resulting in massive cytoplasmic vacuole formation [26] and neurodevelopment [28]. Interestingly, a recent study confirmed that the inflammatory cytokines CSF1 and CXCL10 were upregulated in ZIKV-infected mouse brains at both the protein and messenger RNA levels [36]. In comparison, Zhang et al. also found that innate antiviral response-related genes, including TLR7, MDA5, IRF7, CCL5, MX1, ISG15, IFIT1, and IFIT3, were significantly induced in PBMCs of ZIKV-infected tree shrews at 2 and 6 dpi relative to their expression before infection [37]. In addition, our results indicated that the IL-17A signalling pathway was enriched at 1 dpi, as reported in ZIKV-infected patients previously [38]. Notably, the level of the inflammatory cytokine IL-17A in the serum was not significantly elevated until 3 days after infection (Figure 2H). In general, viral infection usually induces type I interferon signalling. Although no “type I interferon signalling”-related pathway was significantly enriched in ZIKV-infected lungs, we found that some related genes were stimulated. For example, an interferon-stimulated gene, ISG20, was significantly upregulated at 1 and 3 dpi. The tripartite motif (TRIM) family genes TRIM8 and TRIM26, which were significantly upregulated after infection, are also interferon-stimulated genes. We deduced that the type I interferon response might have been stronger at an earlier time point.

In summary, our results demonstrated that ZIKV can infect guinea pigs via aerosols and highlighted the transmission risk associated with exposure to ZIKV aerosols between guinea pigs. However, the transmission efficiency of aerosolized ZIKV under natural conditions remains unknown; thus, further experiments are essential to evaluate the possibility of the transmission of ZIKV and other flavivirus via aerosols.
